# Association between coagulation disorder scores and in-hospital mortality in ARF patients: a retrospective analysis from the MIMIC-IV database

**DOI:** 10.3389/fmed.2023.1184166

**Published:** 2023-05-30

**Authors:** Yuanxing Wu, Guangfa Zhu

**Affiliations:** Department of Pulmonary and Critical Care Medicine, Beijing Anzhen Hospital, Capital Medical University, Beijing Institute of Heart, Lung and Blood Vessel Diseases, Beijing, China

**Keywords:** coagulation disorder score, acute respiratory failure (ARF), Medical Information Mart for Intensive Care IV (MIMIC-IV), in-hospital mortality, anticoagulant

## Abstract

**Introduction:**

Acute respiratory failure (ARF) has a high mortality rate, and currently, there is no convenient risk predictor. The coagulation disorder score was proven to be a promising metric for predicting in-hospital mortality, but its role in ARF patients remains unknown.

**Methods:**

In this retrospective study, data were extracted from the Medical Information Mart for Intensive Care IV (MIMIC-IV) database. Patients diagnosed with ARF and hospitalized for more than 2 days at their first admission were included. The coagulation disorder score was defined based on the sepsis-induced coagulopathy score and was calculated by parameters, namely, additive platelet count (PLT), international normalized ratio (INR), and activated partial thromboplastin time (APTT), based on which the participants were divided into six groups.

**Results:**

Overall, 5,284 ARF patients were enrolled. The in-hospital mortality rate was 27.9%. High levels of additive platelet score, INR score, and APTT score were significantly associated with increased mortality in ARF patients (*P* < 0.001). Binary logistic regression analysis showed that a higher coagulation disorder score was significantly related to the increased risk of in-hospital mortality in ARF patients (Model 2: coagulation disorder score = 6 vs. coagulation disorder score = 0: OR, 95% CI: 7.09, 4.07–12.34, *P* < 0.001). The AUC of the coagulation disorder score was 0.611 (*P* < 0.001), which was smaller than that of sequential organ failure assessment (SOFA) (De-long test P = 0.014) and simplified acute physiology score II (SAPS II) (De-long test *P* < 0.001) but larger than that of additive platelet count (De-long test *P* < 0.001), INR (De-long test *P* < 0.001), and APTT (De-long test *P* < 0.001), respectively. In subgroup analysis, we found that in-hospital mortality was markedly elevated with an increased coagulation disorder score in ARF patients. No significant interactions were observed in most subgroups. Of note, patients who did not administrate oral anticoagulant had a higher risk of in-hospital mortality than those who administrated oral anticoagulant (P for interaction = 0.024).

**Conclusion:**

This study found a significant positive association between coagulation disorder scores and in-hospital mortality. The coagulation disorder score was superior to the single indicators (additive platelet count, INR, or APTT) and inferior to SAPS II and SOFA for predicting in-hospital mortality in ARF patients.

## 1. Introduction

Acute respiratory failure (ARF) implies the inability of the respiratory system to maintain adequate oxygenation of the tissues or remove sufficient carbon dioxide from the tissues (1). It was the most frequent diagnosis during intensive care unit stay (2) and was usually associated with life-threatening complications, high readmission rates, and functional impairment (3, 4). Despite progressive improvements in respiratory support over the recent two decades, in-hospital mortality rate for ARF remains high at ~30% (5). Failure to recognize the clinical deterioration of ARF patients at an early stage is still a significant obstacle (6). Therefore, accurate and early identification of patients with ARF at high risk of death is urgently needed.

Previous studies reported several scoring systems used in ICUs to predict the mortality of patients with ARF. For example, the Simplified Acute Physiology Score-II (SAPS-II) (7, 8) has shown its ability to identify ARF patients at high risk of death. The SOFA score was reported to be significantly associated with mortality in ARF patients (9). However, neither SAPS-II nor SOFA scores are convenient metrics in clinical settings because they are too cumbersome to calculate. The lack of a convenient risk predictor prevents the systematic identification of critically ill ARF patients at high risk of death. It is a major limitation of early intervention or preventive studies of ARF. A reliable and convenient metric for predicting ARF deaths is of urgent need.

The abnormality of blood coagulation parameters was found to be correlated with inflammatory markers such as IL-2R, IL6, IL8, LDH, TNF α, and ferritin, suggesting that the coagulation disorder was an adverse prognostic indicator for ICU patients (10). A recent retrospective study by Long et al. reported the coagulation disorder score, defined by additive platelet count (PLT), international normalized ratio (INR), and activated partial thromboplastin time (APTT) scores, for assessing early coagulation dysfunction, where the coagulation disorder score was found to be a valuable factor in stratifying atrial fibrillation patients with a high risk of in-hospital mortality as well as 90-day mortality (11). The coagulation disorder score has shown its superiority in predicting in-hospital mortality in critically ill congestive heart failure patients (12). Previous studies have found that early preventive anticoagulant therapy prevents severe illness and death in hospitalized patients with ARF, suggesting that coagulation plays an important role in the prognosis of patients with ARF (13). The overactivation of the coagulation system may lead to changes in coagulation indicators (14). Therefore, we hypothesized that the coagulation disorder score might become a promising prognostic biomarker in patients with ARF. In this study, we sought to describe the relationship between the coagulation disorder score and the prognosis of patients with ARF. We also aimed to compare the ability of the coagulation disorder score to predict in-hospital mortality in ARF patients with that of the existing metrics.

## 2. Methods

### 2.1. Population selection criteria

**Figure 1:**
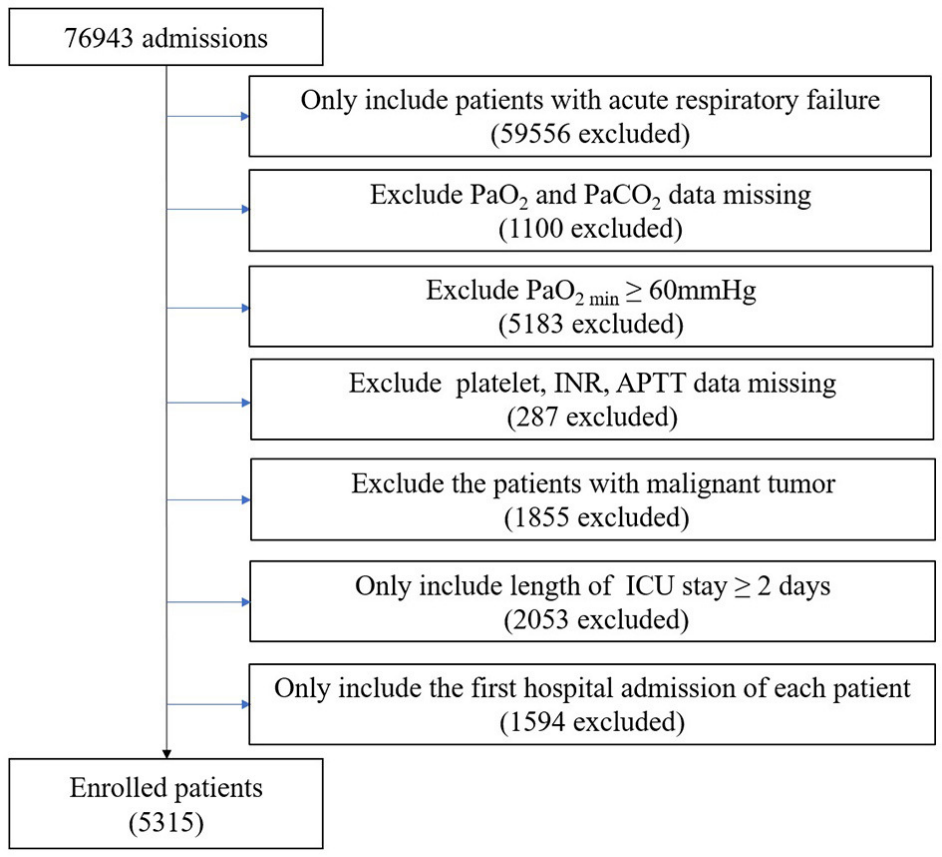
Flow chart of the exclusion and inclusion criteria for selecting subjects. PaO_2_, partial pressure of oxygen; PaCO_2_, partial pressure of carbon dioxide; PaO_2min_, minimum partial pressure of oxygen; INR, international normalized ratio; APTT, activated partial thromboplastin time; ICU, intensive care unit.

Patients diagnosed with acute respiratory failure (ARF) according to ICD-9 Code (J95822, J95821, J960, 51881, 51851, J9602, J9601, and J9600) and hospitalized for more than 2 days at their first admission were included. Patients with the following criteria were excluded: (1) missing data for partial pressure of oxygen (PaO_2_) and partial pressure of carbon dioxide (PaCO_2_); (2) minimum partial pressure of oxygen (PaO_2min_) of ≥ 60 mmHg; (3) missing data for platelet, international normalized ratio (INR) and activated partial thromboplastin time (APTT); and (4) malignant tumor affecting survival. Finally, 5,315 patients were enrolled in the study (Figure 1).

### 2.2. Data extraction

All data were selected from the Medical Information Mart for Intensive Care IV (MIMIC-IV, version 2.1) database, an openly available critical care database, which contains comprehensive and high-quality data on patients admitted to intensive care units (ICUs) at the Beth Israel Deaconess Medical Center between 2008 and 2019 (15, 16). The following data were collected: demographics, vital signs, diagnoses and comorbidities, laboratory parameters, blood gas analysis, treatment, sequential organ failure assessment (SOFA), and simplified acute physiology score II (SAPS II) (details can be found in Supplementary material). All the hematological parameters were obtained by the first blood test after admission within 24 h.

### 2.3. Definition of the coagulation disorder score and coagulopathy endpoints

The coagulation disorder score was defined based on the sepsis-induced coagulopathy (SIC) score or coagulopathy endpoints defined in previous studies (11), which was calculated by adding the points from the following three parts: platelet score (0 point: platelet ≥ 150^*^10^9^/L, 1 point: platelet ≥100 and ≤ 150^*^10^9^/L, 2 point: platelet <100^*^10^9^/L), INR score (0 point: INR < 1.4, 1 point: INR ≥ 1.4, and ≤ 2.6, 2 point: INR > 2.6), APTT score (0 point: APTT ≤ 29 s, 1 point: APTT ≥ 29 s and ≤ 34 s, and 2 point: APTT > 34 s). According to the coagulation disorder score, the participants were divided into six groups: coagulation disorder score = 0 (*n* = 1,201), coagulation disorder score = 1 (*n* = 1,083), coagulation disorder score = 2 (*n* = 1,241), coagulation disorder score = 3 (*n* = 822), coagulation disorder score = 4 (*n* = 547), coagulation disorder score = 5 (*n* = 334), and coagulation disorder score = 6 (*n* = 87). The primary endpoint was in-hospital mortality.

### 2.4. Statistical analysis

Baseline characteristics were summarized as mean ± standard deviation (SD) for normally distributed quantitative data, as median [interquartile range (IQR)] for skewed data, and as number (percentage) for categorical data. Patient characteristics were compared by survival status using an independent-sample *t*-test, the Mann–Whitney U-test, and the chi-squared test, respectively.

Binary logistic regression analysis was used to investigate the relationship between coagulation disorder score and in-hospital mortality, and the results were expressed as odds ratio (OR) and 95% confidence interval (CI). To adjust for relative confounding variables, a multivariate logistic analysis was conducted, including all baseline covariates reported in Table 1, using the stepwise method with removal at a *P*-value of ≥ 0.05. Model 1 was unadjusted. Model 2 was adjusted for age, sex, heart rate, atrial fibrillation, cerebrovascular disease, hypertension, temperature, white blood cell, vasoactive agent, hematocrit, hemoglobin, creatinine, blood nitrogen urea, sodium, mechanical ventilation, acute kidney injury, oral anticoagulant, diabetes, pneumonia, lactate, base excess (BE), maximum partial pressure of carbon dioxide (PaCO_2max_), SOFA, and SAPS II.

**Table 1:** Comparison of baseline data between the survivor and non-survivor groups.

**Parameter**	**All (*n* = 5,315)**	**Survivor (*n* = 3,830)**	**Non-survivor (*n* = 1,485)**	***P*** **-value**
Age (years)	64.2 ± 16.1	62.8 ± 16.2	67.7 ± 15.2	<0.001
Sex, *n* (%)				0.747
Male	2,934 (55.2)	2,120 (55.4)	814 (54.8)	
Female	2,381 (44.8)	1,710 (44.6)	671 (45.2)	
Ethnicity, *n* (%)				<0.001
White	3,342 (62.9)	2,402 (62.7)	940 (63.3)	
Black	581 (10.9)	460 (12.0)	121 (8.1)	
Latino	179 (3.4)	130 (3.4)	49 (3.3)	
Asian	147 (2.8)	108 (2.8)	39 (2.6)	
Others	1,066 (20.1)	730 (19.1)	336 (22.6)	
**Vital signs**				
Systolic blood pressure (mmHg)	121.0 ± 24.5	122.4 ± 24.6	117.4 ± 23.8	<0.001
Diastolic blood pressure (mmHg)	67.6 ± 18.4	68.5 ± 18.3	65.5 ± 18.7	<0.001
Heart rate (beats/min)	92.7 ± 21.0	92.1 ± 20.8	94.4 ± 21.6	<0.001
Respiratory rate (beats/min)	21.5 ± 6.4	21.5 ± 6.4	21.8 ± 6.4	0.143
Temperature (°C)	36.8 ± 0.9	36.9 ± 0.9	36.6 ± 1.0	<0.001
**Diagnoses and comorbidities**, ***n*** **(%)**				
Congestive heart failure	2,374 (44.7)	1,648 (43.0)	726 (48.9)	<0.001
Coronary artery disease	2,065 (38.9)	1,410 (36.8)	655 (44.1)	<0.001
Atrial fibrillation	2,012 (37.9)	1,336 (34.9)	676 (45.5)	<0.001
COPD	1,971 (37.1)	1,455 (38.0)	516 (34.7)	0.030
Pneumonia	3,469 (65.3)	2,464 (64.3)	1,005 (67.7)	0.024
Pulmonary hypertension	378 (7.1)	262 (6.8)	116 (7.8)	0.240
Hypertension	1,810 (34.1)	1,362 (35.6)	448 (30.2)	<0.001
Diabetes	1,859 (35.0)	1,373 (35.8)	486 (32.7)	0.035
Acute kidney injury	4,794 (90.2)	3,371 (88.0)	1,423 (95.8)	<0.001
Chronic kidney disease	1,522 (28.6)	1021 (26.7)	501 (33.7)	<0.001
Sepsis	4,555 (85.7)	3,220 (84.1)	1,335 (89.9)	<0.001
**ARF classification**				0.021
Type I	1,626 (30.6)	1,207 (31.5)	419 (28.2)	
Type II	3,689 (69.4)	2,623 (68.5)	1,066 (71.8)	
**Laboratory parameters**				
White blood cell (10^9^/L)	12.6 ± 7.4	12.3 ± 7.0	13.6 ± 8.0	<0.001
Neutrophil (%)	80.0 ± 11.0	79.7 ± 10.7	80.7 ± 11.5	0.004
Lymphocyte (%)	10.1 ± 7.6	10.4 ± 7.4	9.3 ± 7.9	<0.001
Red blood cell (10^12^/L)	3.6 ± 0.8	3.6 ± 0.8	3.4 ± 0.8	<0.001
Hemoglobin (g/dL)	10.5 ± 2.3	10.6 ± 2.3	10.3 ± 2.2	<0.001
Hematocrit	32.6 ± 6.9	32.8 ± 6.8	31.9 ± 6.9	<0.001
Glucose (mg/dL)	151.2 ± 82.8	151.3 ± 85.7	151.0 ± 74.7	0.892
Creatinine (mg/dL)	1.1 [0.8, 1.9]	1.1 [0.8, 1.8]	1.4 [0.9, 2.2]	<0.001
Blood nitrogen urea (mg/dL)	33.0 ± 25.3	30.9 ± 23.6	38.4 ± 28.7	<0.001
Sodium (mmol/L)	139.0 ± 6.0	139.2 ± 5.8	138.6 ± 6.7	0.001
Potassium (mmol/L)	4.3 ± 0.8	4.2 ± 0.8	4.3 ± 0.8	0.005
Albumin (mmol/L)	3.0 ± 0.6	3.0 ± 0.6	2.8 ± 0.7	<0.001
Platelet (10^9^/L)	209.6 ± 117.4	213.5 ± 115.5	199.6 ± 121.7	<0.001
INR	1.6 ± 1.0	1.5 ± 0.9	1.8 ± 1.2	<0.001
APTT (sec)	39.4 ± 23.4	38.1 ± 22.5	42.8 ± 25.4	<0.001
PT (sec)	17.4 ± 10.3	16.5 ± 9.1	19.6 ± 12.5	<0.001
**Blood gas analysis**				
PaO_2min_	39.1 ± 9.8	39.5 ± 9.7	38.1 ± 10.1	<0.001
PaCO_2max_	60.7 ± 19.6	60.0 ± 18.8	62.5 ± 21.2	<0.001
pH	7.2 ± 0.1	7.3 ± 0.1	7.2 ± 0.1	<0.001
SaO_2_	71.9 ± 19.1	73.6 ± 18.7	68.4 ± 19.5	<0.001
BE	−5 [−10, 0]	−4 [−8, 0]	−8 [−13, −3]	<0.001
Lactate	3.9 ± 3.5	3.3 ± 2.6	5.7 ± 4.7	<0.001
**Treatment**, ***n*** **(%)**				
Oral anticoagulant	1,483 (27.9)	1,231 (32.1)	252 (17.0)	<0.001
Heparin	1,837 (34.6)	1,301 (34.0)	536 (36.1)	0.144
LMWH	475 (8.9)	393 (10.3)	82 (5.5)	<0.001
Vasoactive agent	3,440 (64.7)	2,258 (59.0)	1,182 (79.6)	<0.001
Antibiotics	5,130 (96.5)	3,685 (96.2)	1,445 (97.3)	0.062
Mechanical ventilation	4,164 (78.3)	2,938 (76.7)	1,226 (82.6)	<0.001
ECMO	54 (1.0)	26 (0.7)	28 (1.9)	<0.001
SOFA	8 [6, 12]	8.00 [5, 11]	10.00 [7, 13]	<0.001
SAPS II	42 [34, 53]	40.00 [32, 50]	49.00 [39, 59]	<0.001

Continuous variables were presented as mean ± SD or median (IQR). Categorical variables were presented as numbers (in percentage). *P*-values were calculated using an independent-sample *t*-test, the Mann–Whitney U-test, or the chi-squared test to compare differences in variables between different groups. COPD, chronic obstructive pulmonary disease; ARF, acute respiratory failure; INR, international normalized ratio; APTT, activated partial thromboplastin time; PT, prothrombin time; PaO_2min_, minimum partial pressure of oxygen; PaCO_2max_, maximum partial pressure of carbon dioxide; SaO_2_, oxygen saturation; BE, base excess; LMWH, low molecular weight heparin; ECMO, extracorporeal membrane oxygenation; SOFA, sequential organ failure assessment; SAPA II, simplified acute physiology score II.

The receiver operating characteristic (ROC) curves were drawn, and the areas under the curves (AUCs) of different parameters were compared using the DeLong test.

In subgroup analysis, univariate binary logistic regression was used to investigate the correlation between the coagulation disorder score and in-hospital mortality in different subgroups, and the results were expressed as OR and 95% CI. The *P*-value for interaction was calculated. The forest graph was drawn to demonstrate the results of the subgroup analysis vividly.

All tests were two-sided, and a *P*-value of < 0.05 was considered to be statistically significant. All data analyses were performed by R software (R-project^®^ R Foundation for Statistical Computing, Vienna, Austria, ver. 4.2.1) and MedCalc Software (MedCalc Software Ltd, Antwerpen, Belgium, version. 15.2).

## 3. Results

### 3.1. Patient characteristics

According to the survival state, all participants were divided into two groups: the survivor group (*n* = 3,834) and the non-survivor group (*n* = 1,450). The characteristics of different groups are summarized in Table 1. Compared with the survivor group, patients in the non-survivor group were older, more often Caucasian, had lower blood pressure and temperature but higher heart rate, and more often had a history of congestive heart failure, coronary artery disease, atrial fibrillation, pneumonia, acute kidney injury, chronic kidney disease, sepsis, type II ARF but less often had chronic obstructive pulmonary disease (COPD), hypertension, and diabetes. Moreover, patients in the non-survivor group had higher counts/values of white blood cells, neutrophils, creatinine, blood nitrogen urea, potassium, INR, APTT, prothrombin time (PT) but lower counts/values of lymphocytes, red blood cells, hemoglobin, hematocrit, sodium, albumin, and platelets. Patients in the non-survivor group had higher levels of PaCO_2max_ and lactate but low levels of PaO_2min_, pH, oxygen saturation (SaO_2_), and BE. They also received higher doses of vasoactive agents, prolonged mechanical ventilation, and high-volume extracorporeal membrane oxygenation (ECMO) but lower doses of low molecular weight heparin (LMWH) and oral anticoagulant therapy. SOFA was higher in patients in the non-survivor group compared with those in the survivor group, as was SAPS II.

### 3.2. Association between coagulation disorder score and coagulopathy endpoints

Overall, the in-hospital mortality rate was 27.9%. High levels of the platelet score, INR score, and APTT score were significantly associated with increased mortality in ARF patients, respectively (*P* < 0.001). Moreover, as the coagulation disorder score increased, in-hospital mortality increased significantly (coagulation disorder score = 6 vs. coagulation disorder score = 0: 69.0% vs. 19.4%, *P* < 0.001) (Table 2). In both unadjusted and adjusted logistic regression analyses, we found that a higher coagulation disorder score was significantly associated with the increased risk of in-hospital mortality in ARF patients (Model 1: coagulation disorder score = 6 vs. coagulation disorder score = 0: OR, 95% CI: 9.27, 5.75–15.07, *P* < 0.001; Model 2: coagulation disorder score = 6 vs. coagulation disorder score = 0: OR, 95% CI: 7.09, 4.07–12.34, *P* < 0.001). When considered as a continuous variable in model 1, the coagulation disorder score was associated with a 0.30-fold increase in the risk of mortality (OR, 95% CI: 1.30, 1.25–1.35, *P* < 0.001). In model 2, for each unit increase in the coagulation disorder score, the risk of in-hospital mortality increased by 23% (OR, 95% CI: 1.23, 1.17–1.29, *P* < 0.001) (Table 3).

**Table 2:** Association of the platelet score, INR score, APTT score, and coagulation disorder with in-hospital mortality.

**Parameter**	**All**	**Survivor**	**Non-survivor**	**P-value**
All	5,315	3,830 (72.1)	1,485 (27.9)	
**Platelet score**				<0.001
0 (≥150 10^9^/L)	3,624	2,704 (74.6)	920 (25.4)	
1 (100–150 10^9^/L)	931	669 (71.9)	262 (28.1)	
2 (< 100 10^9^/L)	760	457 (60.1)	303 (39.9)	
**INR score**				<0.001
0 ( ≤ 1.4)	3,515	2,714 (77.2)	801 (22.8)	
1 (1.4–2.6)	1,360	868 (63.8)	492 (36.2)	
2 (>2.6)	440	248 (56.4)	192 (43.6)	
**APTT score**				<0.001
0 (≤ 29 s)	1,751	1,367 (78.1)	384 (21.9)	
1 (29–34 s)	1,408	1,077 (76.5)	331 (23.5)	
2 (>34 s)	2,156	1,386 (64.3)	770 (35.7)	
**Total score**				<0.001
Coagulation disorder score = 0	1,201	968 (80.6)	233 (19.4)	
Coagulation disorder score = 1	1,083	838 (77.4)	245 (22.6)	
Coagulation disorder score = 2	1,241	915 (73.7)	326 (26.3)	
Coagulation disorder score = 3	822	554 (67.4)	268 (32.6)	
Coagulation disorder score = 4	547	336 (61.4)	211 (38.6)	
Coagulation disorder score = 5	334	192 (57.5)	142 (42.5)	
Coagulation disorder score = 6	87	27 (31.0)	60 (69.0)	

INR, international normalized ratio; APTT, activated partial thromboplastin time.

**Table 3:** Association between thecoagulation disorder score and in-hospital mortality.

	**OR (95% CI)**	***P*** **-value**
**Model 1**		
Coagulation disorder score = 0	Reference	
Coagulation disorder score = 1	1.21 [0.99, 1.49]	0.059
Coagulation disorder score = 2	1.48 [1.22, 1.79]	<0.001
Coagulation disorder score = 3	2.01 [1.64, 2.47]	<0.001
Coagulation disorder score = 4	2.61 [2.09, 3.26]	<0.001
Coagulation disorder score = 5	3.07 [2.37, 3.98]	<0.001
Coagulation disorder score = 6	9.23 [5.79, 15.07]	<0.001
Continuous	1.30 [1.25,1.35]	<0.001
**Model 2**		
Coagulation disorder score = 0	Reference	
Coagulation disorder score = 1	1.15 [0.92, 1.44]	0.215
Coagulation disorder score = 2	1.44 [1.16, 1.79]	0.001
Coagulation disorder score = 3	1.78 [1.40, 2.27]	<0.001
Coagulation disorder score = 4	2.26 [1.72, 2.96]	<0.001
Coagulation disorder score = 5	2.20 [1.59, 3.03]	<0.001
Coagulation disorder score = 6	7.09 [4.07, 12.34]	<0.001
Continuous	1.23 [1.17,1.29]	<0.001

Models were derived from binary logistic regression analysis. Model 1: unadjusted. Model 2: adjusted for age, sex, heart rate, atrial fibrillation, cerebrovascular disease, hypertension, temperature, white blood cell, vasoactive agent, hematocrit, hemoglobin, creatinine, blood nitrogen urea, sodium, mechanical ventilation, acute kidney injury, oral anticoagulant, diabetes, pneumonia, lactate, BE, PaCO_2max_, SAPS II, and SOFA. OR, odds ratio; CI, confidence interval; PaCO_2max_, maximum partial pressure of carbon dioxide; BE, base excess; SOFA, sequential organ failure assessment; SAPA II, simplified acute physiology score II.

**Figure 2:**
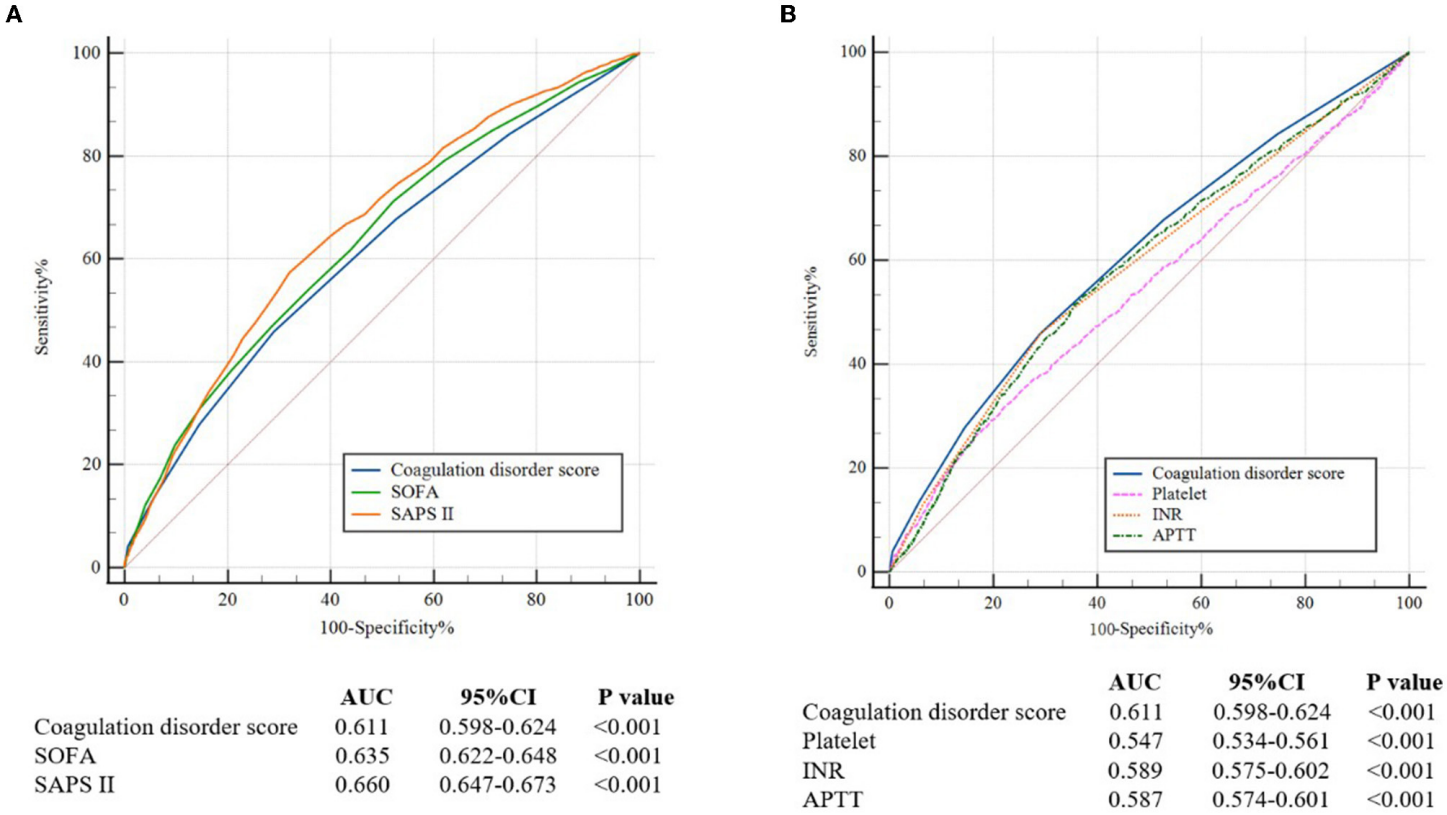
**(A)** The ROC curves for the prediction of in-hospital all-cause mortality of the coagulation disorder score, SOFA, and SAPS II. **(B)** The ROC curves for the prediction of in-hospital mortality of coagulation disorder score, platelet, INR, and APTT. ROC, receiver operating characteristic; AUC, areas under the curve; CI, confidence interval; SOFA, sequential organ failure assessment; SAPA II, simplified acute physiology score II; INR, international normalized ratio; APTT, activated partial thromboplastin time.

Through ROC curves, a certain extent ability of coagulation disorder score to predict in-hospital mortality is shown in Figure 2. The AUC of the coagulation disorder score was 0.611 (*P* < 0.001), which was smaller than that of SOFA (De-long test P = 0.014) and SAPS II (De-long test *P* < 0.001) but larger than that of platelet (De-long test *P* < 0.001), INR (De-long test *P* < 0.001), and APTT (De-long test *P* < 0.001), respectively.

**Figure 3:**
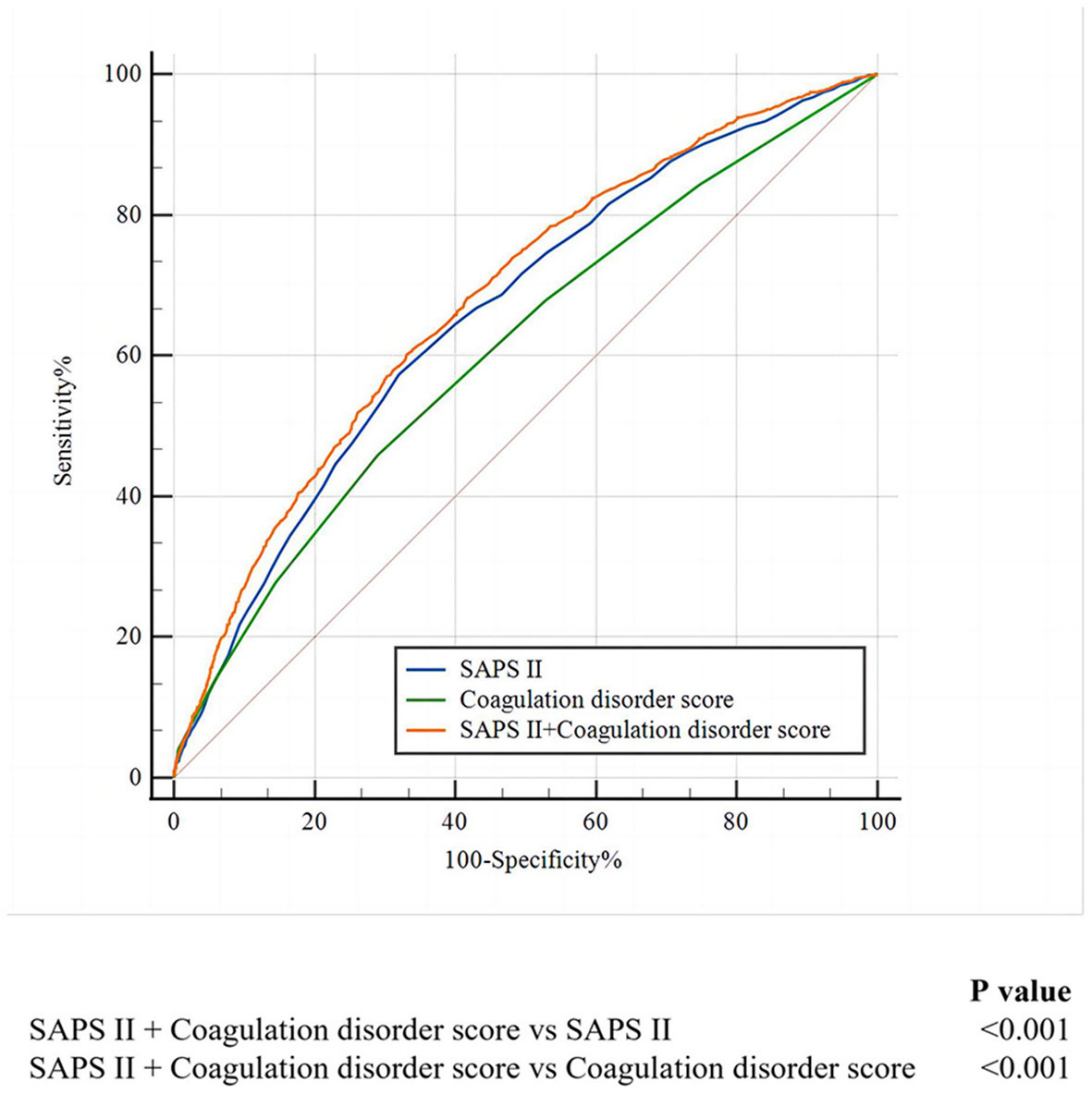
The ROC curves for the prediction of in-hospital mortality of SAFS II, the coagulation disorder score, and the SAPS II + coagulation disorder score. ROC, receiver operating characteristic; SAPA II, simplified acute physiology score II.

As shown in Figure 3, we found that when combining SAPS II with the coagulation disorder score, the AUC of 0.681 was obtained, which was larger than that of SAPS II (De-long test *P* < 0.001) and of coagulation disorder score (De-long test *P* < 0.001).

### 3.3. Subgroup analysis

**Figure 4:**
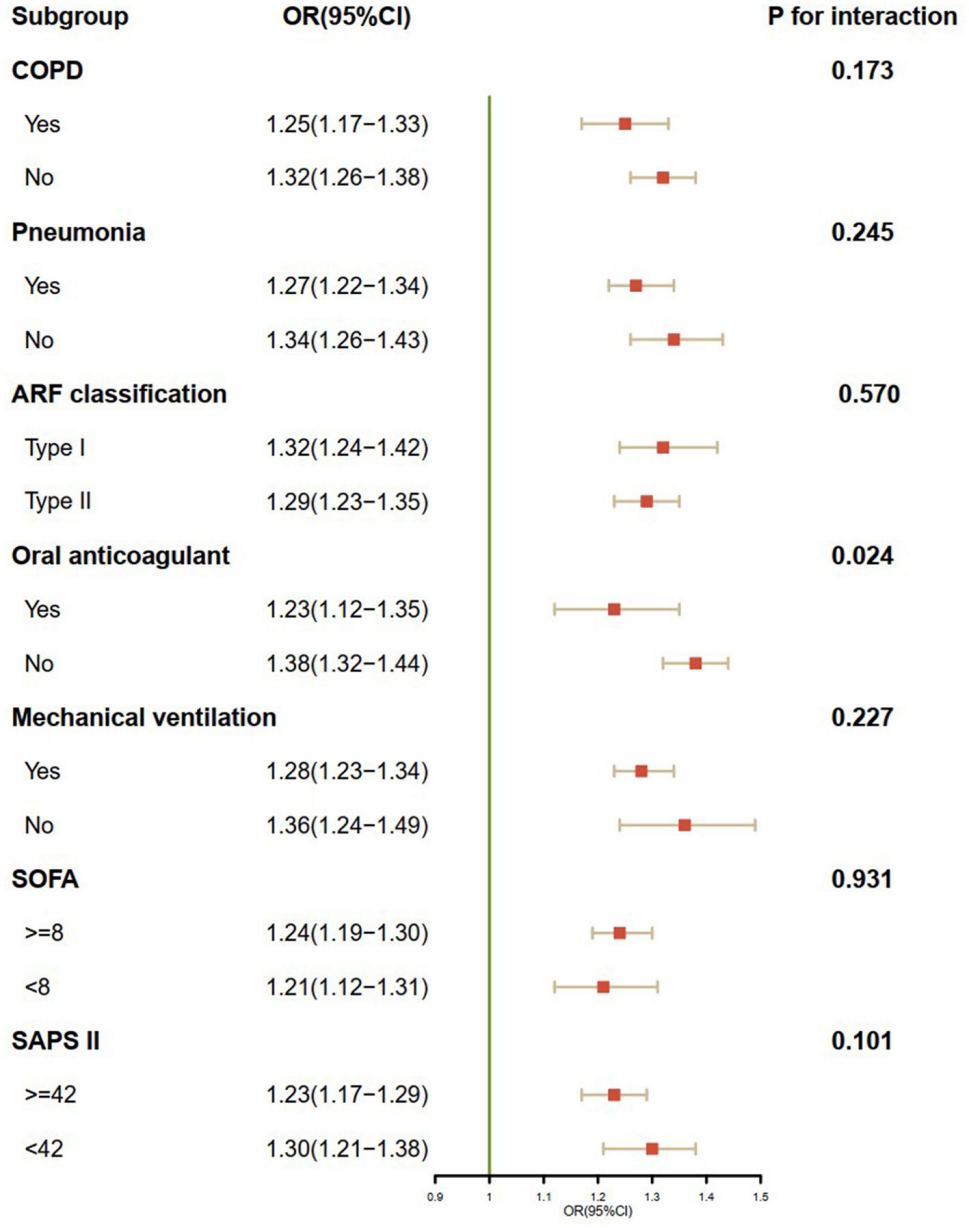
Subgroup analysis of the association between the coagulation disorder score and in-hospital mortality. OR, odds ratio; CI, confidence interval; COPD, chronic obstructive pulmonary disease; ARF, acute respiratory failure; SOFA, sequential organ failure assessment; SAPA II, simplified acute physiology score II.

In all subgroups analyses (Figure 4), we found that in-hospital mortality was markedly elevated with an increased coagulation disorder score in ARF patients. No significant interactions were observed in most subgroups. Patients who were not administered oral anticoagulants had a higher risk of in-hospital mortality than those who were administered oral anticoagulants (P for interaction = 0.024).

## 4. Discussion

In this retrospective study analyzing data from the MIMIC-IV database, we found that a higher coagulation disorder score was significantly associated with an increased risk of in-hospital mortality in ARF patients. The ability of the coagulation disorder score to predict in-hospital mortality in ARF patients was superior to PLT, INR, and APTT but inferior to SOFA and SAPS II. In subgroup analysis, we found that in-hospital mortality was significantly elevated with an increased coagulation disorder score in ARF patients in most subgroups. Patients who were not administered an oral anticoagulant had a higher risk of in-hospital mortality than those who were administered an oral anticoagulant.

Although it is a fairly new indicator introduced in 2021, the coagulation disorder score has already shown its prognostic role in patients with cardiopulmonary dysfunction. Long et al. first reported the coagulation disorder score in assessing early coagulation dysfunction as well as its interaction with atrial fibrillation (11). A retrospective analysis of data from the Medical Information Mart for Intensive Care III (MIMIC-III) database by Tang et al. found a positive association between high coagulation disorder scores and poor prognosis in critically ill congestive heart failure patients (12). The performance of the coagulation disorder score in this study was similar to the reported in previous studies, where a higher coagulation disorder score was found to be significantly associated with the increased risk of in-hospital mortality in ARF patients. In line with the results from the study by Tang et al., compared with the single indicators (PLT, INR, and APTT), the AUC of the coagulation disorder score was larger in our study, suggesting a better ability to predict in-hospital mortality in ARF patients.

We found that the ability of the coagulation disorder score to predict in-hospital mortality in ARF patients was inferior to SOFA and SAPS II. Unlike the multisystem and complicated scoring systems such as SOFA (17) and the SAPS II (18), the coagulation disorder score, which is obtained only through routine admissions, can be used to quickly judge the coagulation system of patients, and the effect is also superior to a single indicator. Although the predictive function of the coagulation disorder score evaluated by ROC was not excellent, the coagulation function evaluated by the coagulation disorder score can be related to outcomes independent of the traditional scores. The combination of the coagulation disorder score and traditional prognostic indicators can improve the predictive accuracy of in-hospital mortality in ARF patients, suggesting that it is a supplement to the traditional prognostic indicators. In clinical practice, a useful risk assessment indicator must balance predictive ability and convenience. The coagulation disorder score is more cost-effective and has a certain predictive ability. It may take the place of SOFA and SAPS II as the available clinical prognostic factor for ARF patients, particularly in circumstances where a more complex score cannot be calculated.

Notably, in subgroup analysis, we found a significant association between the coagulation disorder score and oral anticoagulant administration. Patients who were not administered oral anticoagulants had a higher risk of in-hospital mortality, especially for those with an elevated coagulation disorder score. Oral anticoagulants can change the coagulation system and coagulation function (19), thus changing the coagulation disorder score to a certain extent. In this situation, the change in the coagulation score is a reflection of the therapeutic effect of anticoagulants, which may affect the association between coagulation disorder scores and in-hospital mortality in ARF patients, resulting in the increased risk of in-hospital mortality with an increased coagulation disorder score in patients who were not administered oral anticoagulants.

The pathophysiological process of blood coagulation disorder involves the activation of various immune cells and the production of pro-inflammatory cytokines (20). Pro-inflammatory cytokines are closely associated with abnormal clot formation and play an important role in downregulating important physiological anticoagulant pathways (21). Immunologic studies have revealed that the pro-inflammatory cytokines interleukin 6 (IL-6) and IL-17A are elevated in most patients who die in the hospital (22). In our study, we also found significantly higher baseline white blood cell levels in non-survivors compared with survivors. Due to excessive inflammation, patients with ARF are prone to aggravated hypoxia and thrombosis, which increase the risk of death (23, 24). Therefore, inflammation may be one of the mechanisms that increases the risk of death in ARF patients with coagulation disorders. In addition, vascular endothelial cells (VECs) play an important balancing role between anticoagulation and promoting coagulation (25). Activated VECs produce a large number of adhesion molecules and chemokines, regulating immune cell trafficking and leading to tissue damage and organ failure, which further reduce the patient's survival rate (26). This might also be the reason why the increased risk of in-hospital mortality was associated with a higher coagulation disorder score in ARF patients.

Our study has several strengths. To the best of our knowledge, this study was the first to report the role of the coagulation disorder score in predicting in-hospital mortality in ARF patients. The coagulation disorder score showed superiority in prognostic value and convenience and may be helpful in the decision-making process at the patient's bedside. However, some limitations regarding this study are worth noting. Given the nature of the retrospective study, some residual confounding may not be measured, and possible selection bias cannot be ruled out. Specifically, we failed to clarify the cause of acute respiratory failure and dynamically monitor the coagulation disorder score. In addition, even if the results of this study are promising, external validations on different cohorts will be needed. To date, the coagulation disorder score should be used as a complementary tool for risk stratification.

## 5. Conclusion

In conclusion, this study found a significant positive association between coagulation disorder score and in-hospital mortality, which may provide clinicians with insights into the management of ARF patients. The coagulation disorder score was superior to the single indicators (platelet, INR, or APTT) but inferior to SAPS II and SOFA for predicting in-hospital mortality in ARF patients. Broader validation in terms of the sensitivity and specificity of the coagulation disorder score to predict mortality in different patient populations will be needed.

## Data availability statement

Publicly available datasets were analyzed in this study. This data can be found here: the Medical Information Mart for Intensive Care IV (MIMIC-IV, version 2.2) database at https://doi.org/10.13026/6mm1-ek67.

## Ethics statement

The studies involving human participants were reviewed and approved by the Institutional Review Boards (IRB) of the Massachusetts Institute of Technology (MIT, Cambridge, MA, USA) and Beth Israel Deaconess Medical Center. The patients/participants provided their written informed consent to participate in this study.

## Author contributions

YW and GZ: conceptualization. YW: methodology and writing—original draft. GZ: writing—review and editing. All authors contributed to the article and approved the submitted version.
