# Supplementary material for: Association between coagulation disorder scores and in-hospital mortality in ARF patients: a retrospective analysis from the MIMIC-IV database
Source: Front Med (Lausanne). 2023 May 30;10:1184166. doi: 10.3389/fmed.2023.1184166 (PMC10266267; doi:10.3389/fmed.2023.1184166)
Supplement: Supplementary file 1 [file Data_Sheet_1.pdf]

Supplementary

Data extraction

Following data were collected: demographics (age, sex, ethnicity), vital signs (systolic blood pressure, diastolic blood pressure, heart rate, respiratory rate, temperature), diagnoses and comorbidities (congestive heart failure, coronary artery disease, atrial fibrillation, COPD, pneumonia, pulmonary hypertension, hypertension, diabetes, acute kidney injury, chronic kidney disease, sepsis, ARF classification), laboratory parameter (white blood cell, neutrophil, lymphocyte, red blood cell, hemoglobin, hematocrit, glucose, creatinine, blood nitrogen urea, sodium, potassium, albumin, platelet, INR, APTT, PT), blood gas analysis ($PaO_{2\ min}$, $PaCO_{2\ max}$, pH, SaO_2, BE, lactate), treatment(oral anticoagulant, vasoactive agent, antibiotics, mechanical ventilation, ECMO, heparin, LMWH), SOFA and SAPS II.

$PaO_{2\ min}$ was define as the minimum partial pressure of oxygen during ICU hospitalization. $PaCO_{2\ max}$ was defined as the maximum partial pressure of carbon dioxide during ICU hospitalization.

Vasoactive agent included vasopressin, dopamine, epinephrine, norepinephrine, phenylephrine, dobutamine and milrinone.

Abbreviation: COPD: chronic obstructive pulmonary disease; ARF: acute respiratory failure; INR: international normalized ratio; APTT: activated partial thromboplastin time; PT: prothrombin time; $PaO_{2\ min}$: minimum partial pressure of oxygen; $PaCO_{2\ max}$: maximum partial pressure of carbon dioxide; SaO_2: oxygen saturation; BE: base excess; ECMO: extracorporeal membrane oxygenation; LMWH: low molecular weight heparin; SOFA: sequential organ failure assessment; SAPA II: simplified acute physiology score II; ICU: intensive care unit
